# Early therapy initiation is crucial in chronic inflammatory demyelinating polyneuropathy: prospective multimodal data from the German INHIBIT registry

**DOI:** 10.1007/s00415-024-12860-w

**Published:** 2025-01-07

**Authors:** Aurelian Schumacher, Alina Hieke, Marie Spenner, Fynn Schmitz, Melissa Sgodzai, Rafael Klimas, Jil Brünger, Sophie Huckemann, Jeremias Motte, Anna Lena Fisse, Ralf Gold, Kalliopi Pitarokoili, Thomas Grüter

**Affiliations:** 1 Department of Neurology, St. Josef Hospital, Ruhr University Bochum, 44791 Bochum, Germany; 2 Immune-Mediated Neuropathies Biobank (INHIBIT), Ruhr-University Bochum, Bochum, Germany; 3 Department of Neurology, Evangelic Hospital Lippstadt, 59555 Lippstadt, Germany

**Keywords:** CIDP, Axonal damage, Muscle ultrasound, Therapy

## Abstract

**Background:**

Diagnosing chronic inflammatory demyelinating polyneuropathy (CIDP) can be challenging, leading to delays in initiating therapy. As disability in CIDP is mainly dependent on axonal damage, the impact of delayed immunotherapy remains unclear. We multimodally investigated the clinical outcomes of patients with early CIDP regarding different treatment strategies and time points.

**Methods:**

Patients with CIDP diagnosis within 1 year before study inclusion were systematically selected from the prospective Immune-mediated Neuropathies Biobank (INHIBIT) registry. Clinical and therapeutic data, and findings from nerve conduction study (NCS), and nerve and muscle ultrasound were correlated at inclusion and 12 months later. The patient outcomes were compared between immunotherapies. The effect of timing immunotherapy on clinical outcomes was determined using regression analysis.

**Results:**

In total, 30 patients were included (time from diagnosis to inclusion 22 ± 19 weeks). Low amplitudes of compound muscle potential were significantly associated with pathological spontaneous activity (PSA, *r* = 0.467) and correlated with the Heckmatt scale (*r*_*Sp*_ = 0.391). All three parameters were significantly associated with higher overall disability sum scores (NCS score *r*_*Sp*_ = 0.581, PSA *r* = 0.385, Heckmatt scale *r*_*Sp*_ = 0.472). The delays in initiating therapy resulted in progression of axonal damage (*r*_*Sp*_ = 0.467) and disability (*R*^2^ = 0.200). The combination of first-line therapies led to reduced disability progression (*r* = 0.773), while second-line therapies resulted in improved overall axonal damage (*r* = 0.467).

**Conclusions:**

Axonal damage occurs early and is the main cause of clinical disabilities. Prompt initiation of therapy is crucial to prevent axonal damage and thereby disability progression. A comprehensive therapeutic approach, including a combination of first- or second-line therapies, may improve long-term outcomes.

## Introduction

The diagnosis of chronic inflammatory demyelinating polyneuropathy (CIDP) is primarily based on the clinical phenotype in combination with electrophysiological signs of demyelination, in accordance with the European Academy of Neurology/Peripheral Nerve Society (EAN/PNS) guidelines [1]. The slow progressive onset and necessity for extensive electrophysiological examination make early diagnosis challenging and result in delayed therapy initiation [2–5]. Evaluating disease progression over time is still the standard way to assess disease severity and activity. Even though clinical disability is largely dependent on neurodegeneration [6], the significance of different electrophysiological and ultrasound methods in monitoring of axonal damage and therapy response is unclear, increasing the threshold for escalation therapy. Particularly early disease stages are not well-characterized and the impact of delayed initiation or escalation of immunotherapy on neurodegeneration and disability remains unclear. This is of particular importance because 25% of the patients respond inadequately to first-line therapy [7].

This prospective cohort study aimed to evaluate the effects of delayed therapy initiation on patient outcomes in patients with early CIDP. To this end, the patients with recently diagnosed CIDP were examined using a multimodal approach for markers of axonal damage and inflammation and their therapeutic strategies.

## Methods

### Patient cohort and study design

The patients with CIDP were selectively screened from the prospective Immune-mediated Neuropathies Biobank registry (INHIBIT, Ethics Committee of Ruhr University Bochum, vote no.: 18–6534-BR, registration no.: DRKS00024494) at St. Josef Hospital Bochum, Ruhr University Bochum, based on a diagnosis received within the year prior to inclusion in the registry (baseline). The diagnosis was based on the European Federation of Neurological Societies/Peripheral Nerve Society (EFNS/PNS) diagnostic criteria [8]. Atypical CIDP, including distal acquired demyelinating sensory neuropathy (DADS) and multifocal acquired demyelinating sensory and motor neuropathy (MADSAM), were further differentiated according to Doneddu et al. [9]. Patients with paranodal, anti-myelin-associated glycoprotein, or anti-ganglioside antibodies were excluded. The prospective examinations were conducted at both baseline and the 12-month follow-up (mFU) to collect demographic and clinical data. The patients were treated according to the treating physician’s advice. No treatment intervention was performed on the basis of this study protocol, but information about the therapeutic strategies was collected.

The immunotherapies were separated using first-line therapies (intravenous immunoglobulins [IVIGs], glucocorticosteroids, and plasmapheresis/immunoadsorption). Second-line treatment included all other immunosuppressive or immunomodulatory therapies. Additionally, nerve and muscle ultrasound and nerve conduction study (NCS) results were evaluated. Electromyography was performed only if available during routine diagnostic procedures.

The study was conducted in compliance with the ethical standards laid down in the 1964 Declaration of Helsinki and its later amendments. All the patients provided written informed consent before inclusion in this study.

We used the STROBE cohort checklist when writing our report [10].

### Neurological examinations and clinical scores

All the patients underwent a comprehensive neurological examination. The medical research council (MRC) sum score was used to evaluate the global muscle strength (ranging from 0 to 60 points, covering shoulder abduction, elbow flexion, wrist extension, hip flexion, knee extension, and ankle dorsiflexion). Grip strength was measured using a vigorimeter [11]. To evaluate sensory involvement, the inflammatory neuropathy cause and treatment (INCAT) sensory sum score (ISS) ranging from 0 to 20 was obtained [12]. To access the global degree of disability, we obtained the INCAT overall disability sum score (ODSS, ranging from 0 to 10) [11], inflammatory Rasch-built overall disability scale (I-RODS, ranging from 0 to 48) [13], and modified Rankin scale (mRS, ranging from 0 to 6) [11].

The changes in MRC sum score, grip strength, ISS, ODSS, I-RODS, and mRS scores were rated based on the difference in scores between month 12 after inclusion and baseline (referred to as Δvalue).

### Nerve conduction studies

The NCS were conducted using the Dantec^™^ Keypoint^®^ Focus electromyographic (EMG) device (Natus Medical GmbH, Planegg, Germany). Standard techniques were used for percutaneous supramaximal stimulation and surface electrode in standardized conditions with skin temperatures of at least 33 °C at the palm and 30 °C at the external malleolus. Bilateral NCS were performed on the median (motor and sensory), ulnar (motor and sensory), radial (sensory), tibial (motor), peroneal (motor and sensory), and sural (sensory) nerves in all patients. The NCSs were performed according to the methodology described by Stöhr et al. [14].

The degree of axonal damage in each patient was determined based on the number of nerves that showed a reduction in the amplitude of the distal compound muscle action potential (CMAP) as shown previously [15]. Each nerve was evaluated in binary terms as either affected (below lower limits of normal, LLN) or unaffected (within normal range). The reference values were obtained from Stöhr et al., 6th edition, which provides age-adjusted LLN for CMAP and SNAP amplitudes [16]. Each nerve was evaluated for the presence of a distal conduction block or rapid improvement following immunotherapy induction to minimize the potential effects of demyelination on distal CMAP amplitudes. The scores were assigned as follows: mild (1) for sensory involvement in the lower limbs, moderate (2) for sensorimotor involvement in the lower limbs, severe (3) for sensorimotor involvement in the lower limbs with additional sensory impairment in the upper limbs, and very severe (4) for sensorimotor involvement in both the upper and lower limbs.

To quantify the signs of demyelination, the number of fulfilled EFNS/PNS type 1-criteria [8] were counted.

### Electromyographic examination

Electromyographic examinations were not specifically performed in this study. Data from patients who underwent EMG examination as part of their clinical routine within 6 months prior to or after inclusion in this study were analyzed. All needle EMG tests were performed using uniform equipment (Medtronic four-channel electromyography device; Medtronic, Meerbusch, Germany) and consistent disposable concentric monopolar needle electrodes (50 × 0.46 mm, 0.07 mm^2^ recording area, Value Line DCN, Natus, Ireland) under standardized conditions as described by Stöhr et al. [14]. Examinations of the tibialis anterior, rectus femoris, first dorsal interosseous, biceps brachii, and deltoid muscles were evaluated. Muscle fibrillations and positive sharp waves were examined and consolidated to determine the presence or absence of pathological spontaneous activity (PSA) [17].

### Nerve and muscle ultrasound examination

Ultrasound studies were conducted using an Affiniti 70^®^ (Philips, Hamburg, Germany) by investigators with extensive neuromuscular ultrasound experience. The settings, excluding the depth and focus, were kept constant during all examinations. Ultrasound was performed, as previously described [17–19].

The echogenicity of the muscle tissue was assessed using the Heckmatt score [20] on a scale from 1 (normal muscle echogenicity) to 4 (increased muscle echogenicity). Bilateral examinations of the abductor pollicis brevis, abductor digiti minimi, extensor carpi radialis longus, biceps brachii, triceps brachii, tibialis anterior, and medial head of the gastrocnemius muscles were performed. The evaluation was based on the most severely affected muscles.

Muscle fasciculations were recorded during identical examinations and in the same muscles, as referenced by the Heckmatt score. The evaluation was based on the absolute number of fasciculations observed in the muscle, demonstrating the most fasciculations within a 30-s interval.

To assess nerve swelling, we used the adjusted Bochum ultrasound score (aBUS), as previously described [21], which includes cross-sectional area (CSA) measurements of the median nerve at the forearm and upper arm, ulnar nerve at the forearm and upper arm, radial nerve at the upper arm, and sural nerve at the calf on both sides of the body. The final score was determined by the number of sites with a significantly enlarged CSA (0–6), based on the standard values published by Kerasnoudis et al. [17]. If the same measurement point was enlarged on either side of the body, then only one point was counted.

### Clustering of the diagnostic workup

To differentiate between parameters indicative of axonal damage and demyelination, we separated them as follows: axonal damage was measured by CMAP on the NCS as reflected by the score described above, evidence of PSA on EMG, evidence of fasciculations, and the Heckmatt score on muscle ultrasound. Conversely, the parameters indicative of demyelination included those specified in the ENG diagnostic criteria of EFNS/PNS and aBUS in nerve ultrasound.

### Statistical analyses

Statistical analyses were performed using the IBM SPSS Statistics software (version 26.0.0.0). The ranks of two or more groups were compared using the Mann–Whitney U test or Kruskal–Wallis analysis, respectively. In the case of a significant difference, the effect size r was indicated. Spearman’s correlation coefficient r_Sp_ was used to correlate the two variables. The Friedman test was used to assess the significant differences among multiple related samples. All statistical tests were two-tailed. We used regression analysis to identify the effects of early therapy initiation on the long-term outcomes (I-RODS and MRC sum scores). In all analyses, the threshold for statistical significance was set at *p* < 0.05.

Data are shown as means ± standard deviations for interval-scaled variables or as medians and ranges for ordinal-scaled variables.

Prism 10.3.1 was used to create illustrations.

## Results

### Epidemiological and clinical data

This study included 30 patients (21 male, 9 female) with a mean age at disease onset of 57 ± 11 years. The mean time from the first diagnosis to inclusion was 22 ± 19 weeks and the mean time from therapy initiation to inclusion was 22 ± 20 weeks. In total, 15/30 patients were diagnosed with typical CIDP, 10/30 had DADS, and 5/30 had MADSAM. A total of 4/30 patients had non-insulin-dependent diabetes mellitus.

At the time of study inclusion, the median ODSS was 3 (1–10). When assessing daily functioning, the median I-RODS score was 37.5 (3–48). The mRS score ranged from 1 to 4, with a median of 2. Regarding motor symptoms, the handgrip strength ranged from 0 to 140 kPa, with a mean value of 81.5 kPa. The median MRC sum score was 58 (16.5–60). The sensory involvement was mostly classified as moderate, with a median ISS of 5 (0–15).

At the 1-year follow-up, the patient cohort exhibited a significant improvement in daily activity measurement (ΔI-RODS = 4 ± 9, *p* = 0.041). Furthermore, a nonsignificant improvement was noted in motor symptoms with a mean increase in MRC sum score of 3.5 ± 7.5 points and a mean increase in hand grip strength of 14 ± 28 kPa. The severity of the sensory symptoms did not significantly change throughout the 1-year period. An overview of the clinical scores at baseline and at 12 mFU is shown in Table 1.

**Table 1:** Overview of clinical scores at baseline and 12-month follow-up

	ODSS	I-RODS	mRS	Hand grip strength (kPa)	MRC-SS	ISS
Baseline	3 (1–10)	37.5 (3–48)	2 (1–4)	81.5 (0–140)	58 (16.5–60)	5 (0–15)
12 mFU	2 (0–10)	42 (9–48) *	2 (0–4)	85 (0–130)	59 (27.5–60)	6 (0–14)

*ODSS* Overall Disability Sum Score, *I-RODS* Inflammatory Rasch-Built Overall Disability Scale, *mRS* Modified Rankin Scale, *MRC-SS* Medical Research Council Sum Score, *ISS* INCAT Sensory Sum Score (Inflammatory Neuropathy Cause and Treatment Sensory Sum Score), 12mFU = 12-month follow-up

All values are presented as medians (ranges). *p < 0.05

### Measurement of secondary axonal damage

At baseline, global axonal damage in the NCS was evident using the four-point scale described above, with most patients showing severe (*n* = 12) or very severe (*n* = 12) axonal damage. Age did not correlate with the overall axonal damage score (*p* = 0.8).

In total, 24/30 patients underwent PSA evaluation, of whom 15/24 showed evidence of PSA during the study evaluation period.

The Heckmatt score was assessed in the most severely affected muscle, with a median score of 2 (1–4), indicating increased muscle echogenicity and preserved bone echogenicity. The gastrocnemius muscle was most frequently the most severely affected.

Fasciculations on muscle ultrasound were also evaluated in the most affected muscle, revealing a range of 0–56 fasciculations per 30 s, with a median of nine fasciculations. The gastrocnemius muscle most often showed the highest number of fasciculations per patient.

At the 12-month follow-up, the gastrocnemius muscle remained the most frequently most affected muscle according to the Heckmatt score, whereas the tibialis anterior muscle most often had the highest frequency of fasciculations.

Overall, there were no statistically significant changes in the electrophysiological indicators of axonal damage at 12 mFU compared with baseline in the whole cohort. Similarly, there were no significant changes in muscle echogenicity or the number of fasciculations on ultrasound. Nevertheless, the patients who were included in the study within 1 year of onset demonstrated a significantly greater improvement in the overall score for axonal damage compared to patients included at a later stage (*U* = 35.000, *Z* = −2.741, *p* = 0.006); however, these groups had different treatment strategies and durations before inclusion. A detailed presentation of the variations in clinical scores and technical assessments is shown in Table 2.

**Table 2:** Overview of electrophysiological and ultrasound results at baseline and 12-month follow-up (mFU)

	Axonal damage in NCS	Heckmatt score	Fasciculations per 30 s	Number of met type I criteria	aBUS
Baseline	3 (0–4)	2 (1–4)	9 (0–56)	2 (0–6)	2 (0–6)
12 mFU	3 (0–4)	2 (1–4)	13 (0–57)	4 (1–8)	2 (0–5)

Electromyographic studies were not performed at 12 mFU. All values are shown as medians (ranges). *NCS* Nerve Conduction Studies, *Type I criteria* EFNS/PNS Criteria Supportive of Demyelination, *aBUS* Adjusted Bochum Ultrasound Score

**Fig. 1:**
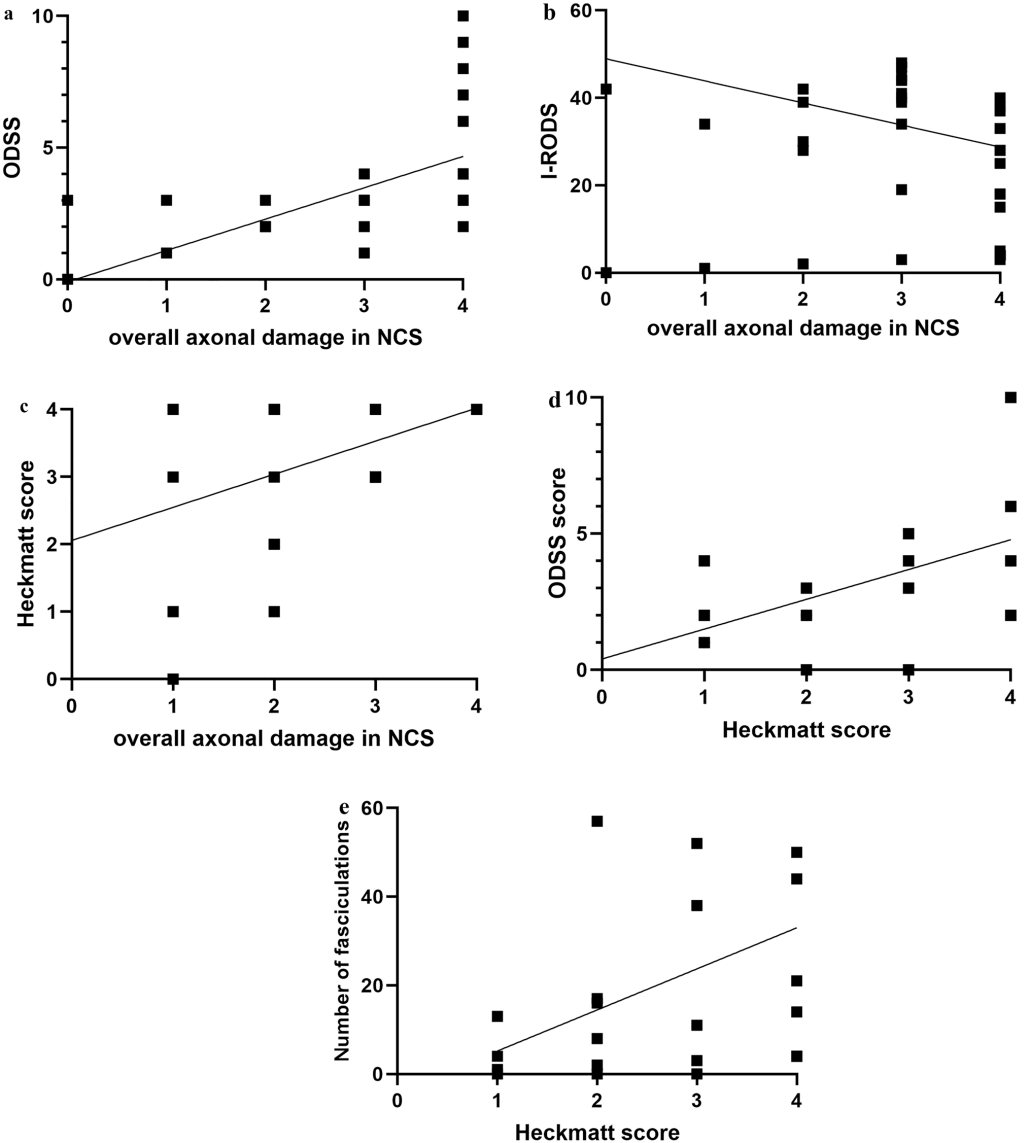
**a** Correlation of overall axonal damage in NCS and ODSS at baseline. A significant positive correlation was observed between axonal damage in NCS and the ODSS score at baseline (*r* = 0.581, *p* < 0.001). NCS = Nerve Conduction Study, ODSS = Overall Disability Sum Score. **b** Correlation of overall axonal damage in NCS and I-RODS at baseline. A significant negative correlation was observed between axonal damage in NCS and the I-RODS score at baseline (*r* = −0.499, *p* = 0.005). NCS = Nerve Conduction Study, I-RODS = Inflammatory Rasch-Built Overall Disability Scale. **c** Correlation of overall axonal damage in NCS and Heckmatt score at baseline. A significant positive correlation was observed between axonal damage in NCS and the Heckmatt score at baseline (*r* = 0.391, *p* = 0.048). NCS = Nerve Conduction Study. **d** Correlation of Heckmatt score and ODSS at 12 mFU. A significant positive correlation was observed between the Heckmatt score and the ODSS score at the 12 mFU. (*r* = 0.472, *p* = 0.042). ODSS = Overall Disability Sum Score, 12mFU = 12-month follow-up. **e** Correlation of Heckmatt score and counted number of fasciculations per 30 s in muscle ultrasound at 12 mFU. A significant positive correlation was observed between the Heckmatt score and the number of fasciculations in ultrasound at the 12 mFU (*r* = 0.507, *p* = 0.027). 12mFU = 12-Month Follow-Up

The presence of PSA on EMG examinations was significantly associated with higher scores on the four-step NCS at baseline (*U* = 32.500, *Z* = −2.290, *r* = 0.467, *p* = 0.022). The overall score of axonal damage in the NCS significantly correlated with the ODSS (r_Sp_ = 0.581, *p* < 0.001, Fig. 1a) and inversely correlated with the I-RODS score (r_Sp_ = −0.499, *p* = 0.005, Fig. 1b) at baseline. This correlation was also observed at the 12 mFU for the I-RODS score (r_Sp_ = −0.505, *p* = 0.02). Comparably, the presence of PSA in the EMG studies was associated with higher ODSS scores at baseline (*U* = 37.500, *Z* = −1.888, *r* = 0.385, *p* = 0.035).

The degree of muscle fibrosis and atrophy was assessed using the Heckmatt scale. Our analysis revealed a significant correlation between axonal damage in the NCS and the Heckmatt score (r_Sp_ = 0.391, *p* = 0.048, Fig. 1c). Although not significant at baseline, the Heckmatt score correlated with the ODSS at the 12 mFU (r_Sp_ = 0.472, *p* = 0.042, Fig. 1d). No association was observed between the Heckmatt score and I-RODS.

At baseline, no statistically significant correlation was noted between the number of fasciculations and the Heckmatt score or axonal damage. However, at the 12 mFU, there was a positive correlation between the number of fasciculations on ultrasound and the Heckmatt score (r_Sp_ = 0.507, *p* = 0.027, Fig. 1e). The number of fasciculations did not correlate with ODSS or I-RODS.

### Measurement of demyelination

When analyzing demyelination markers, a median of 2 (0–6) type I criteria of the EFNS/PNS [8] was met. Systemic nerve enlargement on ultrasound examination was frequently found in our patients, with 72.4% showing two or more enlarged nerve sites. Both markers did not significantly correlate with each other. Over the 1-year disease course, no statistically significant alterations were noted in the electrophysiological signs of demyelination or mean aBUS. An overview of the results at baseline and at 12 mFU is presented in Table 2. Neither parameter correlated with clinical disability as measured by ODSS and I-RODS scores.

### Immunotherapy

**Fig. 2:**
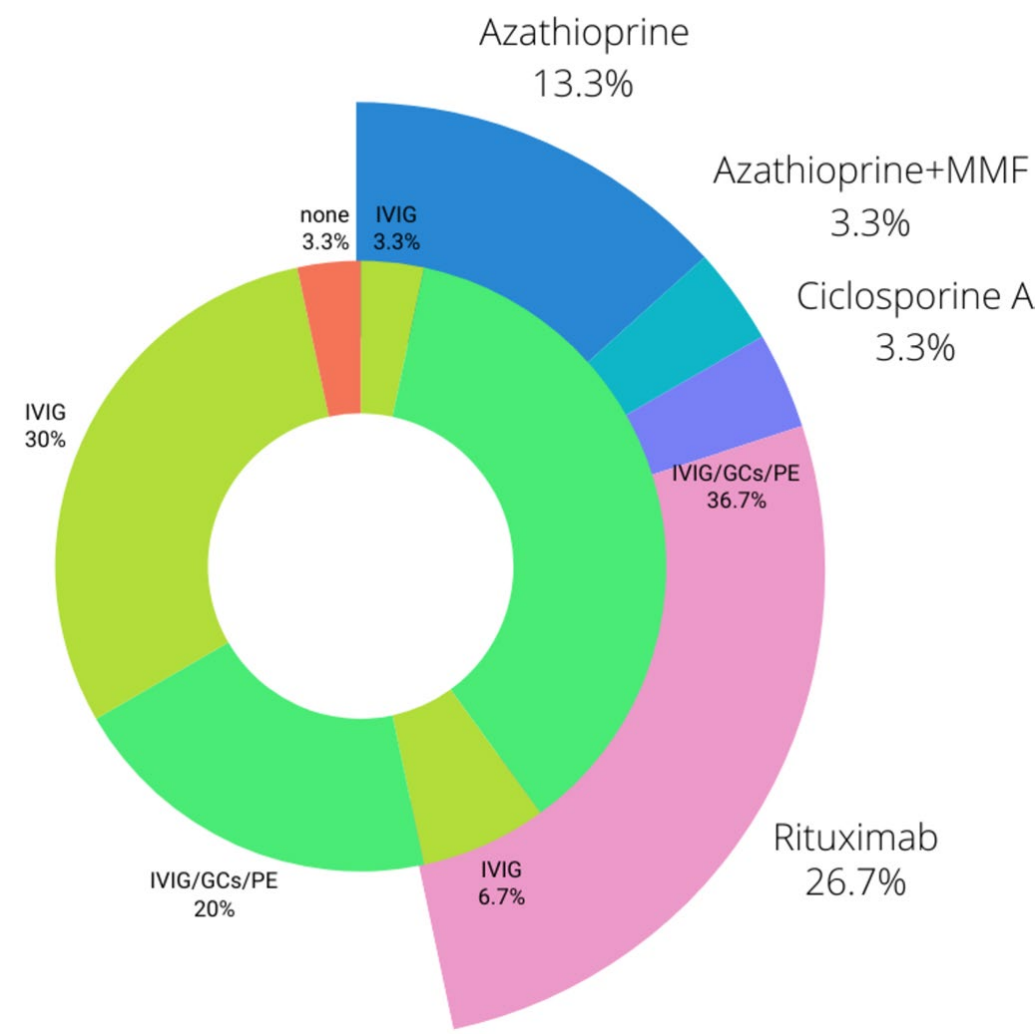
Long-term immunotherapy for all patients in the cohort: the inner circle represents the first-line therapies administered until baseline, with a distinction between those who also received second-line immunotherapy. The outer circle shows the second-line immunotherapy. The order of second-line therapies follows the corresponding first-line therapies. In the cohort, 14/30 (47%) received second-line therapy. First-line therapies included 9 patients who received long-term repetitive intravenous immunoglobulin (IVIG) alone and 6 patients who received long-term repetitive glucocorticosteroids (GCs) alone or in combination with other first-line therapies (IVIG, GCs, or plasma exchange [PE]). Second-line therapies included eight patients receiving rituximab, 4 patients receiving azathioprine, 1 patient receiving azathioprine + mycophenolate mofetil (MMF), and 1 patient receiving cyclosporine A. One patient received no therapy

At study inclusion, a total of 15/30 patients were treated with first-line immunotherapy alone. Of the 15 patients, 9 received IVIG alone, whereas the remaining six received either glucocorticosteroids alone or combined first-line therapy. Fourteen of thirty patients received second-line therapy either in addition to first-line therapy (*n* = 8) or exclusively (*n* = 6) at study inclusion. Among them, rituximab was the most frequently used second-line therapy (*n* = 8). The different treatment strategies are shown in Fig. 2.

One patient who received initial first-line treatment required additional immunosuppressive therapy during the observation period because of signs of severe inflammation and axonal damage. One patient who received initial first-line combination therapy was de-escalated to exclusive IVIG therapy because of rapid symptom improvement. The remaining 27 patients remained on the same regimen for 1 year.

Only one patient did not receive any immunotherapy during the follow-up period because of the side effects of the first-line therapy prior to enrolment.

### Importance of early therapy initiation

**Fig. 3:**
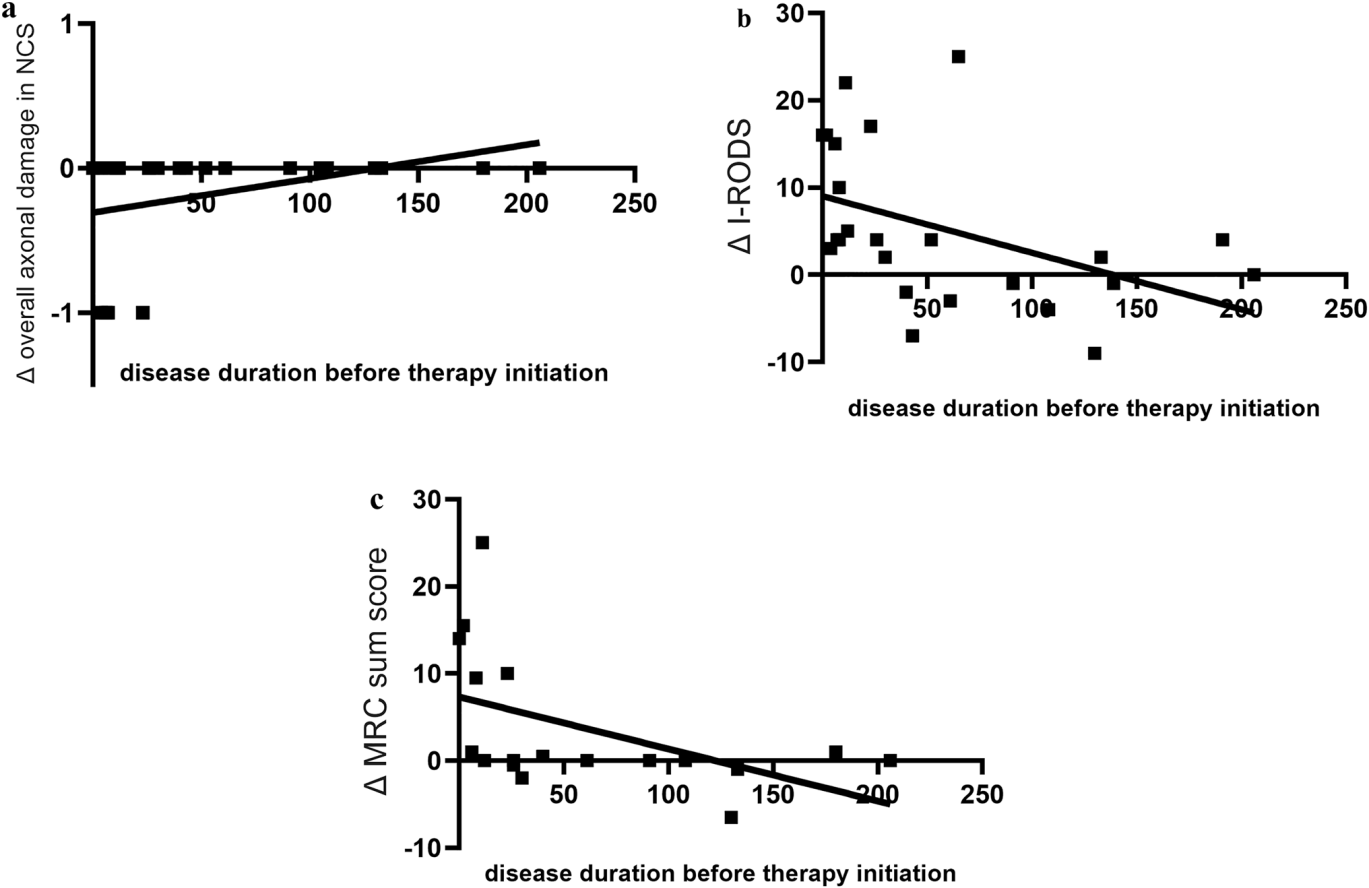
**a** Correlation of disease duration before therapy initiation in weeks and changes in overall axonal damage in NCS from baseline to 12mFU. The significant positive correlation (rSp = 0.467, *p* = 0.025) indicates that longer delays in therapy initiation are associated with reduced improvement in axonal damage. **b** Correlation of disease duration before therapy initiation in weeks and changes in I-RODS score from baseline to 12mFU. Early initiation of therapy is associated with a more significant improvement in I-RODS score (regression coefficient = −0.065 ± 0.028, *R*^2^ = 0.200, *p* = 0.028). **c** Correlation of disease duration before therapy initiation in weeks and changes in MRC sum score from baseline to 12mFU. Early initiation of therapy is associated with a more significant increase in muscle strength (regression coefficient = −0.060 ± 0.026, *R*^2^ = 0.242, *p* = 0.038)

The time between the onset of symptoms and the initiation of therapy significantly correlated with changes in axonal damage in the NCS (r_Sp_ = 0.467, *p* = 0.025, Fig. 3a). Furthermore, regression analysis revealed that early initiation of therapy led to a more pronounced improvement in the I-RODS score (regression coefficient, −0.065 ± 0.028; *R*^2^ = 0.200, *p* = 0.028; Fig. 3b) and muscle strength (MRC sum score, regression coefficient, −0.060 ± 0.026; *R*^2^ = 0.242, *p* = 0.038; Fig. 3c) after 1 year of treatment. A similar trend was observed for the ODSS; however, it did not reach statistical significance (regression coefficient, 0.008 ± 0.006; *p* = 0.152).

### Different therapeutic strategies

In patients who received combination therapy of repetitive IVIGs with steroids or plasma exchange (*n* = 6) during the observation period, the increase in the I-RODS score was significantly higher than that in patients who were administered IVIGs exclusively (*n* = 9) (*U* = 27.500, *Z* = −2.563, *r* = 0.773, *p* = 0.006).

Axonal damage in the NCS improved in 4/30 patients during the 1-year therapy period. All patients had severe or very severe axonal damage at baseline which improved by one step on an ordinal four-step scale. Notably, all four patients received second-line therapy during the observation period, with 50% receiving rituximab. In the entire study cohort, the use of second-line therapy during the observation period was significantly associated with improved overall axonal damage (*U* = 42.000, *Z* = −2.248, *r* = 0.467, *p* = 0.025).

The patient who did not receive immunotherapy was the only one to experienced progression on the four-step scale of axonal damage.

## Discussion

This study presents a comprehensive multimodal longitudinal analysis of a cohort of patients with early CIDP with a focus on diagnostic and therapeutic strategies.

This study highlights the significance of detecting axonal damage as the primary cause of clinical disability because signs of axonal loss, but not demyelination, were associated with clinical disability scores. On one hand, NCS are a well-established tool for diagnosing CIDP and are valid in various studies [22, 23]; however, in some cases, monitoring patients with CIDP solely on NCS can be misleading due to severe axonal damage [4]. On the other hand, the detection of PSA in EMG diagnostics is a valuable marker of progression [24]; however, its invasiveness, associated pain, and contraindications restrict its clinical usage. Muscle ultrasound, owing to its wide availability and noninvasive nature, may be an important complementary tool for monitoring patients with CIDP. The Heckmatt score [20] is correlated with axonal damage in NCS, clinical deterioration, and muscle strength [25], which was confirmed in our study. Furthermore, the Heckmatt score demonstrated in this study also for the first time a correlation with the absolute number of fasciculations in muscle ultrasound. Fasciculations are present in chronic inflammatory demyelinating neuropathies [26] and are often considered signs of active denervation or a manifestation of motor axonal instability, showing recent reinnervation and collateral sprouting [27, 28]. This observation is particularly notable, considering that some studies have indicated that ultrasound may be more effective than electromyography in identifying fasciculations [29]. Our investigations revealed a cohesive pattern linking low CMAPs in the NCS with PSA on EMG examination, higher Heckmatt scores, and muscle fasciculations on ultrasound.

The EAN/PNS criteria for the diagnosis of CIDP recommend nerve ultrasound as a supportive diagnostic tool [1]. The aBUS was developed to differentiate between primary inflammatory and noninflammatory diseases [30]. In our study, we observed frequent inflammation and nerve swelling, which were not associated with clinical disability. Therefore, their prognostic relevance remains unclear. Fibrotic nerve changes may serve as a prognostic factor [31, 32]. Nerve ultrasound can be used to detect changes in the nerve structure and echogenicity. Hyperechogenicity and reduced discrimination of the fascicular structures are negative prognostic factors [31]. Although not part of this study, nerve echogenicity may be a useful tool for monitoring patients with CIDP.

Early initiation of therapy was associated with favorable outcomes. Our findings align with those of Al Zuhairy et al., who retrospectively observed that delayed treatment negatively impacts long-term outcomes [33, 34]. Another study reported that treatment delays may lead to longer overall treatment durations [35]. Furthermore, our study demonstrated that if the first-line therapy fails to lead to a satisfactory response or remission, therapy escalation may be appropriate. A combination of IVIG therapy with other first-line therapeutics, such as corticosteroids and/or plasmapheresis, results in improved disability compared with pure IVIG therapy. These results are consistent with those of another prospective cohort study that favored a combination of first-line therapies [36, 37]. Additionally, second-line therapy reduced axonal damage in some patients. Rituximab was the most frequently used second-line therapy in the current study. Rituximab is associated with favorable outcomes in certain CIDP variants [38–40]. To improve patient outcomes, early therapy escalation by adding an additional first-line or second-line therapy may be necessary. Further clinical studies are required to validate this approach.

This approach is consistent with the principles of early intervention and escalation strategies that have been successfully employed in the management of other autoimmune diseases, such as myasthenia gravis (MG) and multiple sclerosis (MS). In MS, the principle of “no evidence of disease activity” (NEDA) strives for the complete suppression of disease activity from the outset [41, 42]. Similarly, in MG, there is currently a shift towards more aggressive and early-onset therapeutic intervention, with the objective of reducing disease activity [43, 44]. The Food and Drug Administration has recently approved the neonatal Fc receptor antagonist efgartigimod. Despite employing an entirely distinct mechanism of action, efgartigimod has been demonstrated to effectively reduce pathogenic autoantibodies in a manner analogous to rituximab. Both agents have been shown to result in disease improvement in MG [45]. The approval of efgartigimod for CIDP underscores the potential role of second-line therapies [46].

To facilitate early therapeutic escalation, such as second-line therapy, biomarkers are required for prognostic and predictive purposes from the outset, as opposed to evaluating disease progression over time. Elevated serum neurofilament light chain levels in cerebrospinal fluid can serve as a prognostic marker [47]. The detection of corneal inflammatory cells through confocal corneal microscopy may serve as a non-invasive biomarker for the detection of disease activity [48]; however, the current evidence is still evolving.

As axonal damage is the primary cause of clinical disability, further studies are urgently required to identify therapeutic options that can reduce neurodegeneration and support neuroregeneration. Cell culture studies have shown that short-chain fatty acid propionate can provide both neuroprotection and neuroregeneration [49]. Although a case report of a patient with acute motor and sensory axonal neuropathy treated with propionate showed its effectiveness, further clinical studies are required to confirm these findings [50].

### Limitations

First, our study was conducted at a single medical center. Although this approach ensures consistency in practice, center-specific factors, such as patient demographics and disease presentation, may have influenced the outcomes to some extent. This acknowledgement provides a context for interpreting the external applicability of our findings.

Furthermore, the decision to focus on patients with early CIDP may have inadvertently excluded individuals with more severe conditions who have already undergone multiple treatments. This study aimed to investigate a particular subgroup, which may limit the generalizability of our findings.

Additionally, our follow-up duration may not have captured certain long-term effects or shifts in treatment responses. This emphasizes the need for further investigation into the evolution of outcomes beyond the observation period of our study.

We used established criteria such as low distal CMAP, to classify axonal damage. To validate these criteria, we confirmed their relationship with pathological spontaneous activity on electromyography and the Heckmatt score, a marker of muscle atrophy. Previously, overall axonal damage was positively correlated with creatine kinase levels [15]. However, we cannot entirely rule out the possibility that very proximal conduction blocks influenced our results.

Furthermore, single amplitudes may be artificially reduced due to collateral sprouting, potentially affecting the accuracy of axonal damage assessment.

## Conclusion

Early initiation of therapy is crucial for favorable outcomes, as it can prevent further axonal damage and disease progression. Therefore, rapid diagnosis and assessment of the success of therapy using biomarkers are essential.

The potential of axonal damage as a biomarker of disease severity and predictor of further outcomes is highlighted by its effect on functional disability, even in early disease stages. Ultrasound has shown promise as a tool for monitoring CIDP, particularly for identifying axonal damage and serving as a direct indicator of disability.

It is also crucial to adopt a comprehensive and individualized treatment approach when considering available therapeutic options. In severe cases, combinatory first-line therapy and second-line interventions may be necessary. The second-line therapy potentially mitigates axonal damage in specific cases.

## Data Availability

The data collected for this study can be requested upon reasonable request.

## References

[1] Van den Bergh PYK, van Doorn PA, Hadden RDM, Avau B, Vankrunkelsven P, Allen JA et al (2021) European Academy of Neurology/Peripheral Nerve Society guideline on diagnosis and treatment of chronic inflammatory demyelinating polyradiculoneuropathy: report of a joint task force—second revision. Eur J Neurol 28:3556–3583. 10.1111/ene.14959 34327760 10.1111/ene.14959

[2] Bunschoten C, Blomkwist-Markens PH, Horemans A, van Doorn PA, Jacobs BC (2019) Clinical factors, diagnostic delay, and residual deficits in chronic inflammatory demyelinating polyradiculoneuropathy. J Peripher Nerv Syst 24:253–259. 10.1111/jns.12344 31410938 10.1111/jns.12344

[3] Yoon M-S, Chan A, Gold R (2011) Standard and escalating treatment of chronic inflammatory demyelinating polyradiculoneuropathy. Ther Adv Neurol Disord 4:193–200. 10.1177/1756285611405564 21694819 10.1177/1756285611405564 PMC3105635

[4] Allen JA (2020) The misdiagnosis of CIDP: a review. Neurol Ther 9:43–54. 10.1007/s40120-020-00184-6 32219701 10.1007/s40120-020-00184-6 PMC7229131

[5] van Doorn IN, Eftimov F, Wieske L, van Schaik IN, Verhamme C (2024) Challenges in the early diagnosis and treatment of chronic inflammatory demyelinating polyradiculoneuropathy in adults: current perspectives. Ther Clin Risk Manag 20:111–126. 10.2147/TCRM.S360249 38375075 10.2147/TCRM.S360249 PMC10875175

[6] Barnett MH, Mathey E, Kiernan MC, Pollard JD (2016) Axonal damage in central and peripheral nervous system inflammatory demyelinating diseases: common and divergent pathways of tissue damage. Curr Opin Neurol 29:213–221. 10.1097/WCO.0000000000000334 27058223 10.1097/WCO.0000000000000334

[7] Fisse AL, Motte J, Grüter T, Sgodzai M, Pitarokoili K, Gold R (2020) Comprehensive approaches for diagnosis, monitoring and treatment of chronic inflammatory demyelinating polyneuropathy. Neurol Res Pract 2:42. 10.1186/s42466-020-00088-8 33324942 10.1186/s42466-020-00088-8 PMC7722337

[8] Van den Bergh PYK, Hadden RDM, Bouche P, Cornblath DR, Hahn A, Illa I et al (2010) European Federation of Neurological Societies/Peripheral Nerve Society guideline on management of chronic inflammatory demyelinating polyradiculoneuropathy: report of a joint task force of the European Federation of Neurological Societies and the Peripheral Nerve Society - first revision. Eur J Neurol 17:356–363. 10.1111/j.1468-1331.2009.02930.x 20456730 10.1111/j.1468-1331.2009.02930.x

[9] Doneddu PE, Cocito D, Manganelli F, Fazio R, Briani C, Filosto M et al (2019) Atypical CIDP: diagnostic criteria, progression and treatment response. Data from the Italian CIDP Database. J Neurol Neurosurg Psychiatry 90:125–32. 10.1136/jnnp-2018-318714 30297520 10.1136/jnnp-2018-318714

[10] von Elm E, Altman DG, Egger M, Pocock SJ, Gøtzsche PC, Vandenbroucke JP et al (2008) The Strengthening the Reporting of Observational Studies in Epidemiology (STROBE) statement: guidelines for reporting observational studies. J Clin Epidemiol 61:344–349. 10.1016/j.jclinepi.2007.11.008 18313558 10.1016/j.jclinepi.2007.11.008

[11] Merkies I, Schmitz P, van der Meche FGA, Samijn J, van Doorn PA (2002) Clinimetric evaluation of a new overall disability scale in immune mediated polyneuropathies. J Neurol Neurosurg Psychiatry 72:596–601. 10.1136/jnnp.72.5.596 11971045 10.1136/jnnp.72.5.596 PMC1737884

[12] Merkies IS, Schmitz PI, van der Meché FG, van Doorn PA (2000) Psychometric evaluation of a new sensory scale in immune-mediated polyneuropathies. Inflammatory Neuropathy Cause and Treatment (INCAT) Group. Neurology 54:943–9. 10.1212/wnl.54.4.943 10690990 10.1212/wnl.54.4.943

[13] van Nes SI, Vanhoutte EK, van Doorn PA, Hermans M, Bakkers M, Kuitwaard K et al (2011) Rasch-built Overall Disability Scale (R-ODS) for immune-mediated peripheral neuropathies. Neurology 76:337–345. 10.1212/WNL.0b013e318208824b 21263135 10.1212/WNL.0b013e318208824b

[14] Stöhr M, Pfister R, Reilich P (2022) Klinische Elektromyographie und Neurographie: Lehrbuch und Atlas. Kohlhammer Verlag, Stuttgart

[15] Grüter T, Motte J, Bulut Y, Kordes A, Athanasopoulos D, Fels M et al (2022) Axonal damage determines clinical disability in chronic inflammatory demyelinating polyradiculoneuropathy (CIDP): a prospective cohort study of different CIDP subtypes and disease stages. Eur J Neurol 29:583–592. 10.1111/ene.15156 34687104 10.1111/ene.15156

[16] Stöhr M, Pfister R (2014) Klinische elektromyographie und neurographie - Lehrbuch und Atlas. 6th ed. Koehlhammer, Stuttgart. 10.17433/978-3-17-028373-2

[17] Kerasnoudis A, Pitarokoili K, Behrendt V, Gold R, Yoon M-S (2014) Multifocal motor neuropathy: correlation of nerve ultrasound, electrophysiological, and clinical findings. J Peripher Nerv Syst JPNS 19:165–174. 10.1111/jns5.12067 24862982 10.1111/jns5.12067

[18] Pitarokoili K, Schlamann M, Kerasnoudis A, Gold R, Yoon M-S (2015) Comparison of clinical, electrophysiological, sonographic and MRI features in CIDP. J Neurol Sci 357:198–203. 10.1016/j.jns.2015.07.030 26227829 10.1016/j.jns.2015.07.030

[19] Fisse AL, Pitarokoili K, Motte J, Gamber D, Kerasnoudis A, Gold R et al (2019) Nerve echogenicity and intranerve CSA variability in high-resolution nerve ultrasound (HRUS) in chronic inflammatory demyelinating polyneuropathy (CIDP). J Neurol 266:468–475. 10.1007/s00415-018-9158-3 30554264 10.1007/s00415-018-9158-3

[20] Heckmatt JZ, Leeman S, Dubowitz V (1982) Ultrasound imaging in the diagnosis of muscle disease. J Pediatr 101:656–660. 10.1016/s0022-3476(82)80286-2 7131136 10.1016/s0022-3476(82)80286-2

[21] Brünger J, Motte J, Grüter T, Mork H, Bulut Y, Carolus A et al (2022) Nerve ultrasound distinguishes non-inflammatory axonal polyneuropathy from inflammatory polyneuropathy with secondary axonal damage. Front Neurol. 10.3389/fneur.2021.809359 35153986 10.3389/fneur.2021.809359 PMC8831897

[22] Rajabally YA, Nicolas G, Piéret F, Bouche P, den Bergh PYKV (2009) Validity of diagnostic criteria for chronic inflammatory demyelinating polyneuropathy: a multicentre European study. J Neurol Neurosurg Psychiatry 80:1364–1368. 10.1136/jnnp.2009.179358 19622522 10.1136/jnnp.2009.179358

[23] Breiner A, Brannagan TH III (2014) Comparison of sensitivity and specificity among 15 criteria for chronic inflammatory demyelinating polyneuropathy. Muscle Nerve 50:40–46. 10.1002/mus.24088 24338746 10.1002/mus.24088

[24] Grüter T, Motte J, Fisse AL, Bulut Y, Köse N, Athanasopoulos D et al (2020) Pathological spontaneous activity as a prognostic marker in chronic inflammatory demyelinating polyneuropathy. Eur J Neurol 27:2595–2603. 10.1111/ene.14476 32794258 10.1111/ene.14476

[25] Fisse AL, Fiegert S, Stoykova Z, Brünger J, Athanasopoulos D, Grüter T et al (2021) Increased muscle echointensity correlates with clinical disability and muscle strength in chronic inflammatory demyelinating polyneuropathy. Eur J Neurol 28:1698–1705. 10.1111/ene.14716 33404183 10.1111/ene.14716

[26] Pegat A, Boisseau W, Maisonobe T, Debs R, Lenglet T, Psimaras D et al (2020) Motor chronic inflammatory demyelinating polyneuropathy (CIDP) in 17 patients: clinical characteristics, electrophysiological study, and response to treatment. J Peripher Nerv Syst 25:162–170. 10.1111/jns.12380 32364302 10.1111/jns.12380

[27] Mills KR (2005) The basics of electromyography. J Neurol Neurosurg Psychiatry 76:ii32-5. 10.1136/jnnp.2005.069211 15961866 10.1136/jnnp.2005.069211 PMC1765694

[28] Desai J, Swash M (1997) Fasciculations: what do we know of their significance? J Neurol Sci 152:s43–s48. 10.1016/S0022-510X(97)00243-8 9419053 10.1016/s0022-510x(97)00243-8

[29] Duarte ML, Iared W, Oliveira ASB, dos Santos LR, Peccin MS (2020) Ultrasound versus electromyography for the detection of fasciculation in amyotrophic lateral sclerosis: systematic review and meta-analysis. Radiol Bras 53:116–121. 10.1590/0100-3984.2019.0055 32336828 10.1590/0100-3984.2019.0055 PMC7170585

[30] Kerasnoudis A, Pitarokoili K, Gold R, Yoon M-S (2015) Bochum ultrasound score allows distinction of chronic inflammatory from multifocal acquired demyelinating polyneuropathies. J Neurol Sci 348:211–215. 10.1016/j.jns.2014.12.010 25534358 10.1016/j.jns.2014.12.010

[31] Härtig F, Ross M, Dammeier NM, Fedtke N, Heiling B, Axer H et al (2018) Nerve ultrasound predicts treatment response in chronic inflammatory demyelinating polyradiculoneuropathy—a prospective follow-up. Neurotherapeutics 15:439–451. 10.1007/s13311-018-0609-4 29435815 10.1007/s13311-018-0609-4 PMC5935640

[32] Niu J, Zhang L, Fan J, Liu J, Ding Q, Guan Y et al (2022) Nerve ultrasound may help predicting response to immune treatment in chronic inflammatory demyelinating polyradiculoneuropathy. Neurol Sci 43:3929–3937. 10.1007/s10072-022-05882-7 35061135 10.1007/s10072-022-05882-7

[33] Al-Zuhairy A, Jakobsen J, Moldovan M, Krarup C (2022) Axonal loss at time of diagnosis as biomarker for long-term disability in chronic inflammatory demyelinating polyneuropathy. Muscle Nerve 66:715–722. 10.1002/mus.27722 36217677 10.1002/mus.27722 PMC9828077

[34] Al-Zuhairy A, Jakobsen J, Krarup C (2021) Early axonal loss predicts long-term disability in chronic inflammatory demyelinating polyneuropathy. Clin Neurophysiol 132:1000–1007. 10.1016/j.clinph.2020.12.017 33581994 10.1016/j.clinph.2020.12.017

[35] Rajabally YA, Min YG, Nazeer KK, Englezou C (2024) Treatment response amplitude and timing in chronic inflammatory demyelinating polyneuropathy with routine care: Study of a UK cohort. Eur J Neurol 31:e16399. 10.1111/ene.16399 38980202 10.1111/ene.16399 PMC11414796

[36] Bus SRM, Zambreanu L, Abbas A, Rajabally YA, Hadden RDM, de Haan RJ et al (2021) Intravenous immunoglobulin and intravenous methylprednisolone as optimal induction treatment in chronic inflammatory demyelinating polyradiculoneuropathy: protocol of an international, randomised, double-blind, placebo-controlled trial (OPTIC). Trials 22:155. 10.1186/s13063-021-05083-1 33608058 10.1186/s13063-021-05083-1 PMC7894234

[37] Adrichem ME, Bus SR, Wieske L, Mohammed H, Verhamme C, Hadden R et al (2020) Combined intravenous immunoglobulin and methylprednisolone as induction treatment in chronic inflammatory demyelinating polyneuropathy (OPTIC protocol): a prospective pilot study. Eur J Neurol 27:506–513. 10.1111/ene.14096 31571349 10.1111/ene.14096 PMC7028131

[38] Querol L, Nogales-Gadea G, Rojas-Garcia R, Diaz-Manera J, Pardo J, Ortega-Moreno A et al (2014) Neurofascin IgG4 antibodies in CIDP associate with disabling tremor and poor response to IVIg. Neurology 82:879–886. 10.1212/WNL.0000000000000205 24523485 10.1212/WNL.0000000000000205 PMC3959751

[39] Querol L, Rojas-García R, Diaz-Manera J, Barcena J, Pardo J, Ortega-Moreno A et al (2015) Rituximab in treatment-resistant CIDP with antibodies against paranodal proteins. Neurol Neuroimmunol Neuroinflammation. 10.1212/NXI.0000000000000149 10.1212/NXI.0000000000000149 PMC4561230 26401517

[40] Chaganti S, Hannaford A, Vucic S (2022) Rituximab in chronic immune mediated neuropathies: a systematic review. Neuromuscul Disord NMD 32:621–627. 10.1016/j.nmd.2022.05.013 35672205 10.1016/j.nmd.2022.05.013

[41] Wong B, Cahill J, Rizvi S (2013) Moving towards a cure for MS: increased immunosuppression and striving for no evidence of disease activity (NEDA). R I Med J 2018(101):26–29 29490321

[42] Simpson A, Mowry EM, Newsome SD (2021) Early aggressive treatment approaches for multiple sclerosis. Curr Treat Options Neurol 23:19. 10.1007/s11940-021-00677-1 34025110 10.1007/s11940-021-00677-1 PMC8121641

[43] Utsugisawa K, Nagane Y, Akaishi T, Suzuki Y, Imai T, Tsuda E et al (2017) Early fast-acting treatment strategy against generalized myasthenia gravis. Muscle Nerve 55:794–801. 10.1002/mus.25397 27603432 10.1002/mus.25397 PMC5484288

[44] Farrugia ME, Goodfellow JA (2020) A practical approach to managing patients with myasthenia gravis—opinions and a review of the literature. Front Neurol 11:604. 10.3389/fneur.2020.00604 32733360 10.3389/fneur.2020.00604 PMC7358547

[45] Howard JF, Bril V, Burns TM, Mantegazza R, Bilinska M, Szczudlik A et al (2019) Randomized phase 2 study of FcRn antagonist efgartigimod in generalized myasthenia gravis. Neurology 92:e2661–e2673. 10.1212/WNL.0000000000007600 31118245 10.1212/WNL.0000000000007600 PMC6556100

[46] Efficacy, Safety, and Tolerability of Efgartigimod in Patients with Chronic Inflammatory Demyelinating Polyneuropathy: Results from the ADHERE Trial (PL5.002) | Neurology n.d. https://www.neurology.org/doi/10.1212/WNL-.0000000000206324. Accessed 9 July 2024.

[47] Godelaine J, De Schaepdryver M, Bossuyt X, Van Damme P, Claeys KG, Poesen K (2021) Prognostic value of neurofilament light chain in chronic inflammatory demyelinating polyneuropathy. Brain Commun 3:fcab018. 10.1093/braincomms/fcab018 33796853 10.1093/braincomms/fcab018 PMC7991223

[48] Motte J, Grüter T, Fisse AL, Bulut Y, Stykova Z, Greiner T et al (2021) Corneal inflammatory cell infiltration predicts disease activity in chronic inflammatory demyelinating polyneuropathy. Sci Rep 11:15150. 10.1038/s41598-021-94605-7 34312451 10.1038/s41598-021-94605-7 PMC8313721

[49] Grüter T, Mohamad N, Rilke N, Blusch A, Sgodzai M, Demir S et al (2023) Propionate exerts neuroprotective and neuroregenerative effects in the peripheral nervous system. Proc Natl Acad Sci U S A 120:e2216941120. 10.1073/pnas.2216941120 36669102 10.1073/pnas.2216941120 PMC9942889

[50] Yoon M-S, Pitarokoili K, Sturm D, Haghikia A, Gold R, Fisse AL (2018) Treatment of an acute motor and sensory axonal neuropathy with propionate in a 33-year-old male. Ther Adv Neurol Disord 11:1756286418809580. 10.1177/1756286418809580 30542375 10.1177/1756286418809580 PMC6236647

